# Quantitative discrimination of *Aggregatibacter actinomycetemcomitans* highly leukotoxic JP2 clone from non-JP2 clones in diagnosis of aggressive periodontitis

**DOI:** 10.1186/1471-2334-12-253

**Published:** 2012-10-11

**Authors:** Akihiro Yoshida, Oum-Keltoum Ennibi, Hideo Miyazaki, Tomonori Hoshino, Hideaki Hayashida, Tatsuji Nishihara, Shuji Awano, Toshihiro Ansai

**Affiliations:** 1 Division of Community Oral Health Science, Kyushu Dental College, Kitakyushu, Japan; 2 Département de Parodontologie, Faculté de Médecine Dentaire, Université Mohammed V Souissi, Rabat, Morocco; 3 Department of Oral Health Science, Niigata University Graduate School of Medical and Dental Science, Niigata, Japan; 4 Department of Pediatric Dentistry, Nagasaki University Graduate School of Biomedical Sciences, Nagasaki, Japan; 5 Department of Preventive Dentistry, Nagasaki University Graduate School of Biomedical Sciences, Nagasaki, Japan; 6 Division of Infections and Molecular Biology, Kyushu Dental College, Kitakyushu, Japan

**Keywords:** *Aggregatibacter actinomycetemcomitans*, Aggressive periodontitis, JP2, Non-JP2, qPCR, Quantification

## Abstract

**Background:**

*Aggregatibacter actinomycetemcomitans* is the etiological agent of periodontitis, and there is a strong association between clone JP2 and aggressive periodontitis in adolescents of African descent. The JP2 clone has an approximately 530-bp deletion (∆530) in the promoter region of the *lkt*/*ltx* gene, which encodes leukotoxin, and this clone has high leukotoxic activity. Therefore, this clone is very important in aggressive periodontitis. To diagnose this disease, culture methods and conventional PCR techniques are used. However, quantitative detection based on qPCR for the JP2 clone has not been developed due to genetic difficulties. In this study, we developed a qPCR-based quantification method specific to the JP2 clone.

**Methods:**

Based on our analysis of the DNA sequence of the *lkt*/*ltx* gene and its flanking region, we designed a reverse primer specific for the ∆530 deletion border sequence and developed a JP2-specific PCR-based quantification method using this primer. We also analyzed the DNA sequence of the ∆530 locus and found it to be highly conserved (97–100%) among 17 non-JP2 strains. Using the ∆530 locus, we designed a qPCR primer–probe set specific to non-JP2 clones. Next, we determined the numbers of JP2 and non-JP2 clone cells in the periodontal pockets of patients with aggressive periodontitis.

**Results:**

The JP2-specific primers specifically amplified the genomic DNA of the *A*. *actinomycetemcomitans* JP2 clone and did not react with other bacterial DNA, whereas the non-JP2 specific primers reacted only with *A*. *actinomycetemcomitans* non-JP2 clones. Samples from the 88 periodontal sites in the 11 patients with aggressive periodontitis were analyzed. The bacterial cell numbers in 88 periodontal sites ranged from 0 to 4.8 × 10^8^ (mean 1.28 × 10^7^) for JP2 clones and from 0 to 1.6 × 10^6^ for non-JP2 clones (mean 1.84 × 10^5^). There were significant differences in the JP2 cell number between a clinical attachment level (CAL) ≤6 mm and a level ≥7 mm (*p* < 0.01). Our new qPCR-based JP2- and non-JP2-specific quantitative detection assay is applicable to the diagnosis of aggressive periodontitis with *A*. *actinomycetemcomitans*.

**Conclusions:**

We successfully developed a quantitative and discriminative PCR-based method for the detection of *A*. *actinomycetemcomitans* JP2 and non-JP2 clones. This technique will contribute to future analyses of the quantitative relationship between this organism and aggressive periodontitis.

## Background

Periodontitis is an infectious disease comprising a complex group of inflammatory conditions that eventually result in the loss of the teeth-supporting tissues [1]. Several systemic diseases are thought to be associated with periodontal disease [2-4]. Of the various forms of periodontitis, aggressive periodontitis is characterized by an early age of onset, with molar and incisor teeth the first ones affected in localized forms [5-7]. Studies have revealed an association between this disease and the presence of *Aggregatibacter actinomycetemcomitans*, a Gram-negative, facultative anaerobic coccobacillus [8-10]. *A*. *actinomycetemcomitans* isolated from aggressive periodontitis in adolescents of African descent living in different parts of the world are genetically homogeneous and belong to a single clone called JP2 [11-15]. The high pathogenicity of this clone has been supported by various epidemiological studies of people of African descent [8,16]. The pathogenicity of this clone is characterized by increased leukotoxin production. The highly leukotoxic JP2 clone of *A*. *actinomycetemcomitans* serotype b is characterized by a 530-bp deletion (∆530) in the promoter region of the *lkt*/*ltx* gene operon, which encodes the leukotoxin, resulting in increased leukotoxin production [17-20]. This clone shows pronounced racial tropism, as it has been isolated almost exclusively from adolescent periodontitis patients of West and Northwest African descent [14,18]. Haubek *et al.* reported that the JP2 clone is an important etiological agent in the initiation of periodontal attachment loss in adolescents [8,21,22].

Consequently, the accurate diagnosis of this disease is based on detecting the JP2 clone from periodontal lesions in patients with aggressive periodontitis. Previously, subgingival plaque cultures were used to analyze the association between the presence of the JP2 clone and aggressive periodontitis [23]. Since this is time consuming, detection using cultures is not suitable for rapid diagnosis. Molecular genetics techniques, which are relatively rapid, including conventional PCR and other molecular-based techniques, have been used to detect the JP2 clone from subgingival plaque [24,25]. However, these are qualitative methods, and no completely quantitative molecular-based detection technique for the JP2 clone has been reported [26]. Furthermore, a quantitative analysis of the *A*. *actinomycetemcomitans* JP2 clone and the severity of aggressive periodontitis has not been done, although positive relationships between the numbers of *Porphyromonas gingivalis* and *Treponema denticola* cells and the severity of adult periodontitis have been reported [27-29].

In this study, we developed a quantitative, discriminative detection method specific to *A*. *actinomycetemcomitans* JP2 and non-JP2 clones using qPCR (real-time PCR). This is the first report of quantitative detection specific to the *A*. *actinomycetemcomitans* JP2 and non-JP2 clones. Additionally, the relationship between the number of cells and the severity of this disease was examined. This method should contribute to clarifying the quantitative relationship between the JP2 clone and aggressive periodontitis.

## Results

### Analysis of the *A*. *actinomycetemcomitans lktC* locus for primer design

**Figure 1:**
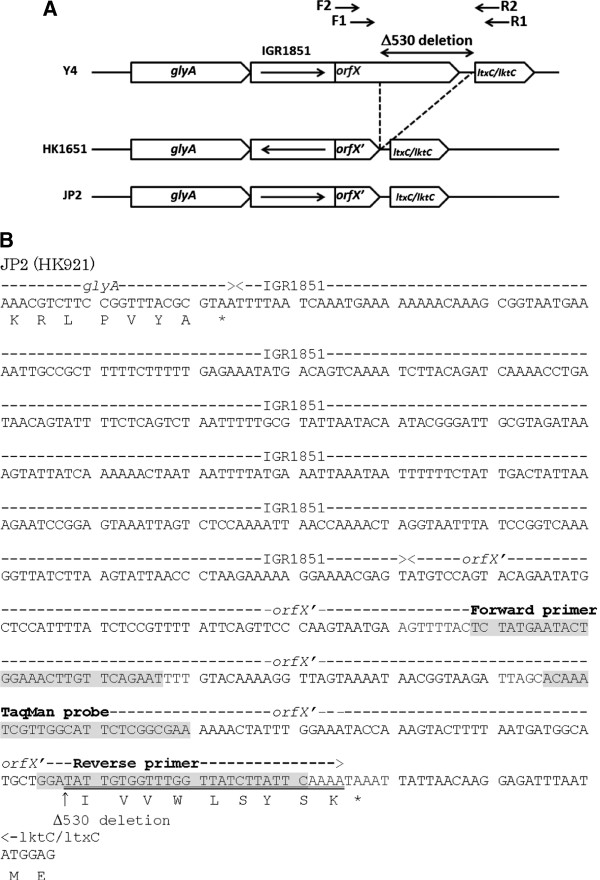
**Comparison of (A) the genetic organization of JP2 clones and non-JP2 strains and (B) the nucleotide sequences of the *glyA *gene and *orfX *(*orfX'*)*-lktC *locus of the JP2 strain.** (**A**) The ∆530-bp deletion of the JP2 strain was identified. F1 and R1, Aa orfX3'F1 and Aa lktC5'R1; F2 and R2, Aa orfX3'F2 and Aa lktC5'R2. (**B**) The nucleotide sequence of the JP2-specific primer–probe set. The arrow indicates the ∆530-bp deletion. The double underlined amino acid sequence (IVVWLSYSK) overlaps the 3' locus of *orfX*

Figure 1 shows the gene cluster and nucleotide sequence of the region upstream from the *ltxC/lktC* locus. Downstream from the *glyA* gene, IGR1851 (IGR: non-coding region of genomic DNA, Gene ID: AA02808) was identified. The nucleotide sequence of IGR1851 of HK1651 is on the strand opposite that of Y4 and JP2 (Figure 1A). In the Y4 strain, the region upstream from *ltxC/lktC* contains a 762-bp open-reading frame (ORF). The ORF of the JP2 clone, named *orfX'*, contains an approximately 530-bp deletion compared with *orfX* of the non-JP2 strain. The 31-bp of sequence at the 3’ end following the deletion is common to both *orfX* and *orfX’* (Figure 1B, double underlined). Therefore, we designed an antisense primer specific to the JP2 clone at the border of the 3' region of *orfX'* and this 31-bp 3' locus of *orfX* (Figure 1B).

### Sequencing analysis of the ∆530 region for designing non-JP2 specific primers

To evaluate the specificity of the ∆530 locus in the primer design, we analyzed the PCR products using primer pairs designed outside the ∆530 locus. Initially, we performed PCR analysis using the primer pair Aa orfX3'F1 and Aa lktC5'R1 (Figure 1A, Table 1). Of the 18 non-JP2 strains, seven were positive using this primer set (723 bp, Table 2). To amplify the ∆530 locus of the other strains, primer pair Aa orfX3'F2 and Aa lktC5'R2 was also designed (Figure 1A, Table 1). Using these primers, in 10 of 11 non-JP2 strains, except for NCTC 9710, the locus 3’ of *orfX* was amplified (809 bp, Table 2). The DNA sequences of the ∆530 locus of 17 strains were analyzed using these amplicons. Using the DNA sequence data, homology analysis was performed among these 17 strains (Additional file 1: Table S2). Of the 17 non-JP2 strains, the ∆530 region consisted of 528 bp for strains Y4 and ATCC 29522 and 531 bp for the other strains (Additional file 1: Table S2). The homologies were between 96.987 and 100%. Therefore, the ∆530 locus was highly conserved among non-JP2 strains. Furthermore, the homology search revealed that the nucleotide sequences of the ∆530 locus were specific to the non-JP2 strains (blastn, data not shown). Therefore, we considered the ∆530 locus to be a nucleotide marker for non-JP2 strains and designed a qPCR primer set and hydrolysis probe specific to the non-JP2 strains in this region (Table 1).

**Table 1:** Oligonucleotide primers and hydrolysis probes used in this study

**Primers or Probes**	**Sequence**	**Gene**	**Size**
Primer			
JP2-F3	5’-TCT ATG AAT ACT GGA AAC TTG TTC AGA AT-3’	*orfX’*	151 bp^d^
JP2-R2	5’-GAA TAA GAT AAC CAA ACC ACA ATA TCC-3’	*orfX’*	
Non-JP2F	5’-CGC AAG TGC CAT AGT TAT CCA CT-3’	*orfX* (∆530 region)	145 bp^e^
Non-JP2R	5’-TCG TCT GCG TAA TAA GCA AGA GAG-3’	*orfX* (∆530 region)	
Aa orfX3’F1	5’-AAA TCG TTG GCA TTC TCG -3’	*orfX*	723 bp^e^
Aa lktC5’R1	5’-CAA AGG AGA ATT TGC CCA -3’	*lktC*	
Aa orfX3’F2	5’-CCG TTT TAT TCA GTT CCC -3’	*orfX*	809 bp^e^
Aa lktC5’R2	5’-TGC CCA TAA CCA AGC CAC -3’	*lktC*	
Hydrolysis probes^a, b, c^			
JP2	5’-FAM-ACA AAT CGT TGG CAT TCT CGG CGA A-TAMRA-3’	*orfX’*	
Non-JP2	5’-FAM-ATA TTG TAG ACA TCG CCC-MGB-3’	*orfX* (∆530 region)	

^a^FAM, 6-carboxyfluorescein.

^b^TAMRA, 6-carboxytetramethylrhodamine.

^c^MGB, minor groove binder.

^d^calculated in JP2 strain.

^e^calculated in Y4 strain.

**Table 2:** **Primer specificity for the *Aggregatibacter actinomycetemcomitans *strains**

**Strain**	**Serotype**	**JP2/non-JP2**	**Amplification with primer pair:**^**a**^		**Primer pairs for Δ530 locus analysis**^**b**^**(for non-JP2)**	**Source or reference**
			**JP2F3-JP2R2**	**NonJP2F- NonJP2R**		
ATCC 29523	a	non-JP2	-	+	1	ATCC^c^
SUNYaB 75 (ATCC 43717)	a	non-JP2	-	+	1	SUNYaB^d^
TN-1	a	non-JP2	-	+	2	Nishihara^e^
Y4 (ATCC 43718)	b	non-JP2	-	+	1	Socransky^f^
JP2 (HK921)	b	JP2	+	-	-	Kilian^g^
HK 1199	b	JP2	+	-	-	Kilian
HK 1519	b	JP2	+	-	-	Kilian
HK 1611	b	JP2	+	-	-	Kilian
HK 1612	b	JP2	+	-	-	Kilian
HK 1651	b	JP2	+	-	-	Kilian
HK 1709	b	non-JP2	-	+	1	Kilian
ATCC 29522	b	non-JP2	-	+	2	ATCC
ATCC 29524	b	non-JP2	-	+	1	ATCC
HK 916	c	non-JP2	-	+	1	Kilian
SUNYaB 67 (ATCC 43719)	c	non-JP2	-	+	1	SUNYaB
NCTC 9709	c	non-JP2	-	+	2	NCTC^h^
NCTC 9710	c	non-JP2	-	+	none	NCTC
3381	d	non-JP2	-	+	2	Asikainen^i^
IDH 781	d	non-JP2	-	+	2	Asikainen
OMZ 534	e	non-JP2	-	+	2	Gmür^j^
OMZ 541	e	non-JP2	-	+	2	Gmür
OMZ 546	e	non-JP2	-	+	2	Gmür
NUM 5005	f	non-JP2	-	+	2	Takada^k^
NUM 4039	g	non-JP2	-	+	2	Takada

^a^ +, positive amplification with a band of the expected size; -, no amplification observed.

^b^1, Aa orfX3’F1 and Aa lktC5’R1; 2, Aa orfX3’F2 and Aa lktC5’R2 (Table 1); none, not 1 or 2s.

^c^ATCC, American Type Culture Collection, Rockville, Md.

^d^SUNYaB, State University of New York at Buffalo, NY.

^e^Nishihara, T. Nishihara, Kyushu Dental College, Kitakyushu, Japan.

^f^Socransky, S. Socransky, Forsyth Dental Center, Mass.

^g^Kilian, M. Kilian, University of Aarhus, Aarhus, Denmark.

^h^NCTC, National Collection of Type Cultures, London, England.

^i^Asikainen, S. Asikainen, University of Helsinki, Helsinki, Finland.

^j^Gmür, R. Gmür, University of Zurich, Zurich, Switzerland.

^k^Takada, K. Takada, Nihon University School of Dentisry at Matsudo, Chiba, Japan.

### Specificity and sensitivity of the JP2- and non-JP2-specific primers

Using these primer pairs and probe, we confirmed the primers’ specificity by checking their reactivity with various strains of *A*. *actinomycetemcomitans* and other oral bacteria (Tables 2 and Additional file 2: Table S1). The JP2-specific primers specifically amplified the genomic DNA of the *A*. *actinomycetemcomitans* JP2 clone and did not react with other bacterial DNA, whereas the non-JP2 specific primers reacted only with *A*. *actinomycetemcomitans* non-JP2 clones (Tables 2 and Additional file 2: Table S1). The sensitivity of this TaqMan assay for *A*. *actinomycetemcomitans* JP2 and non-JP2 clones was evaluated. Serially diluted chromosomal DNA of *A*. *actinomycetemcomitans* JP2 and non-JP2 clones was used to evaluate the lower detection limits. The lower detection limit was 2.19 pg/mixture for the JP2 clone and 2.24 pg/mixture for the non-JP2 clone. The standard curves for linear regressions between the threshold cycle (Ct) values and corresponding colony forming units (CFU) were obtained. The standard curves for *A*. *actinomycetemcomitans* JP2 and non-JP2 were *Y* = -3.548*X* + 40.96, R^2^ = 1.00 and *Y* = −3.501*X* + 36.91, R^2^ = 1.00 (*Y* = Ct, *X* = CFU), respectively (data not shown). The detection limits of the assays determined with serial dilutions of *A*. *actinomycetemcomitans* JP2 and Y4 genomic DNA were 4 to 4 × 10^6^ copies (for JP2, corresponding to 4 to 4 × 10^6^ CFU) and 11 to 1.1 × 10^7^ copies (for Y4, corresponding to 11 to 1.1 × 10^7^ CFU), respectively (data not shown).

### Inhibitory effect of oral specimens on the assay (spike analysis)

The presence of inhibitors in the oral specimens was assessed using lysates spiked with dental plaque (ca. 1 g [wet weight] per mixture, to mimic subgingival plaque) without the *A*. *actinomycetemcomitans* JP2 clone. The presence of dental plaque caused negligible inhibition of the real-time PCR amplification (data not shown). A similar spike experiment was performed using the non-JP2 specific primers and dental plaque without *A*. *actinomycetemcomitans* strains. Dental plaque had no inhibitory effects on the assay (data not shown).

### Quantification of the JP2 and non-JP2 clones from subgingival plaque samples

Samples from the 88 periodontal sites (31 incisors, 44 first molars, 13 premolars) in the 11 patients with aggressive periodontitis were analyzed. Nine of the 11 patients were positive for JP2 clones, and three of these nine were also positive for non-JP2 clones. Two patients were negative for both clones. Forty-six sites were positive for JP2 clones (52.3%); of these, nine sites were positive for both JP2 and non-JP2 clones (10.2%). One site was positive only for non-JP2 clones (1.1%). Neither clone was detected at 39 sites (44.3%). The bacterial cell numbers in 88 periodontal sites ranged from 0 to 4.8 × 10^8^ (mean 1.28 × 10^7^) for JP2 clones and from 0 to 1.6 × 10^6^ for non-JP2 clones (mean 1.84 × 10^5^).

### Correlation between periodontal status and the number of JP2 cells

All samples were divided into two groups based on the CAL, and the mean number of JP2 clone cells was determined. There was a significant difference in the number between groups with CAL ≤ 6 mm (mean 4.74 mm) and that with CAL ≥7 mm (mean 9.12 mm) (*p* < 0.01, Table 3). The analysis of non-JP2 was not clear due to the small number of non-JP2 positive samples (*n* = 10, data not shown).

**Table 3:** The number of JP2 clones in 88 subgingival plaque samples and the grouping in terms of the CAL

**CAL**	**Mean ± SD of bacterial cell number**	***P***^***a***^
≤ 6 mm (n= 31)	3.79×10^5^ ± 1.58×10^5^	0.003
≥ 7 mm (n= 57)	1.92×10^7^ ± 8.35×10^7^	

^*a*^ Mann–Whitney test.

## Discussion

Studies have shown that highly leukotoxic *A*. *actinomycetemcomitans* strains, *i.e.*, the JP2 clone, are associated with aggressive periodontitis [8]. A cross-sectional study of Moroccan adolescents found a strong association between the presence of the JP2 clone and periodontal attachment loss [23]. Furthermore, a population-based longitudinal study found an increased risk of developing periodontal attachment loss when the JP2 clone of *A*. *actinomycetemcomitans* was present in the subgingival plaque [8,30]. However, these investigations were based on qualitative evaluations of the JP2 strain, *i.e.*, the relationship between the presence or absence of JP2 and the severity of aggressive periodontitis. The quantitative relationship between the number of JP2 cells and the severity of aggressive periodontitis had not been discussed prior to this report. A previous investigation reported a differentiation and quantification method for *A*. *actinomycetemcomitans* JP2 and 652 strains [26]. However, this differentiation system was based on amplicon size differences and requires agarose gel electrophoresis or melting-curve analysis to distinguish these strains. Therefore, the assay is not a primer-specificity-based method. Consequently, we developed both JP2-clone- and non-JP2-clone-specific quantification methods based on primer specificity and the qPCR technique.

**Figure 2:**
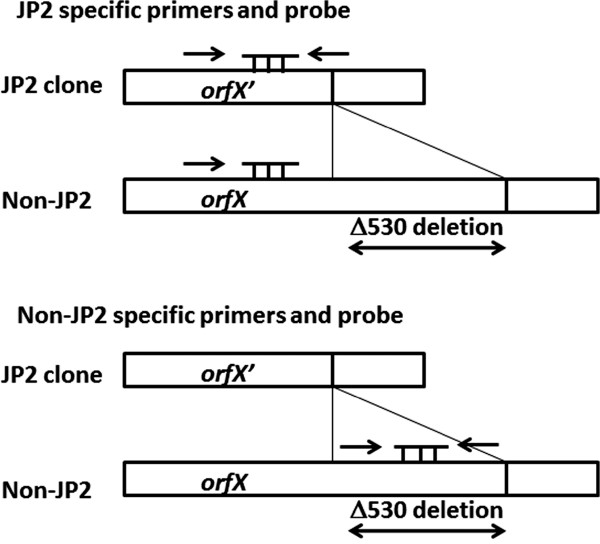
**Designation of the oligonucleotide primers and probes specific to the JP2 clone (above) and non-JP2 strains (below).** For the JP2-specific amplification, the sense primer and hydrolysis probe are complementary to *orfX'* (for JP2 clone) and *orfX* (for non-JP2 strains). The antisense primer was designed to be complementary to the border of the 3' end of *orfX'* for the specific amplification of JP2 clones. For non-JP2-specific selection, the sense and antisense primers and TaqMan probe were designed to be complementary to the *orfX* ∆530 region

First, we analyzed the nucleotide sequences of the promoter region of the *ltxC/lktC* gene. For this purpose, we analyzed the PCR products using the primer pairs Aa orfX3'F1 and Aa lktC5'R1 or Aa orfX3'F2 and Aa lktC5'R2. However, the *orfXltxC*/*lktC* locus was not amplified only in the NCTC 9710 strain using these primers. In addition, using the primer pair Aa orf3’F1-Aa lktC5’R2 and Aa orf3’F2-Aa lktC5’R1, the *orfXltxC*/*lktC* locus in the NCTC 9710 strain was not amplified by PCR. We postulated that the DNA sequences of these primers differ from those of the NCTC 9710 strain, but this is no more than a supposition. As described previously, the most dramatic variation is an approximately 530-bp deletion in the JP2 leukotoxin promoter relative to the non-JP2 strain [17,18,20]. The 530-bp deletion removes a DNA segment starting in the middle of *orfX*, a 762-bp ORF found in Y4 and ATCC 33384, and continues to a position 25 bp 5' to the *ltxC/lktC* start codon. Additionally, IGR1851 was identified between the *glyA* and *ltxC/lktC* genes. According to the Oralgen database ( http://www.oralgen.lanl.gov/_index.html) of the Los Alamos National Laboratory, the nucleotide sequence of IGR1851 of HK1651 is on the strand opposite that of Y4 and JP2 (Figure 1A). Consequently, we designed a sense primer and hydrolysis probe inside *orfX'* and the antisense primer border of the 3' region of *orfX'* and 3' of *orfX* (Figure 1B, double underlined). For the JP2 clone, the sense and antisense JP2-specific primers anneal to the appropriate loci on *orfX'* (Figure 2). For the non-JP2 clone, however, the sense primer anneals to *orfX'*, while the antisense primer is not able to anneal to the 3' border of *orfX'* due to the lack of 530-bp deletion (Figure 2). This primer design successfully amplified only the JP2 clone. Conversely, non-JP2 specific oligonucleotide primers were designed inside the ∆530-bp locus. To analyze the conservation and specificity of the ∆530-bp locus, we sequenced it, revealing that the ∆530-bp locus is highly conserved among non-JP2 strains (ranging from 96.987 to 100%). The non-JP2-specific primer sets successfully amplified non-JP2 strains, but not the JP2 strain. As shown in Table 2, the specificities of these assays were evaluated. Additionally, we examined the effect of non-target DNA on this assay, and the influence of the co-presence of non-target DNA was negligible (data not shown). This spiking experiment showed that this characteristic is suited to detection in oral specimens. Next, we evaluated the sensitivities of this assay. Using serially diluted chromosomal DNA (see results), we found that the sensitivities of these assays were consistent with previous studies [27,31]. Using this assay, we detected and quantified both JP2 and non-JP2 clones from subgingival plaque samples from adolescents.

In this study, 48 (54.5%) of the 88 periodontal sites (in nine of 11 individuals) were positive for JP2 clones. Previous studies reported that the prevalence of the JP2 strain in Moroccan adolescents was 50 of 428 (11.7%) individuals [8] and 43 of 365 (11.8%) individuals [30]. Our study subjects were aggressive periodontitis patients, whereas the other studies included subjects with healthy periodontium. Therefore, those data cannot strictly be compared with our results. Additionally, the JP2 and non-JP2 clones were detected using conventional PCR in those studies. Seki *et al.* applied the loop-mediated isothermal amplification (LAMP) method for detecting the JP2 clone in subgingival plaque from Moroccan adolescents and found that 48 of 72 samples (66.7%) were JP2 positive [25]. The LAMP technology was very sensitive for the JP2 clone, and the detection limit was 10 genome copies, whereas that with conventional PCR was 100 copies [25,32,33]. These findings are identical to the sensitivity of qPCR, and the detection rate was similar to our result (54.5%). In this study, we quantified the number of cells of the JP2 and non-JP2 clones. We also analyzed the relationship between cell number and CAL. There was a significant difference in JP2 cell number between group with a CAL ≤6 mm and that with ≥7 mm (*p* < 0.01). The mean JP2 cell number of the CAL >7 mm group was more than 50 times that of the CAL <6 mm group. Previously, several population-based studies demonstrated that the presence of the *A*. *actinomycetemcomitans* JP2 clone increased the risk of progression of CAL over a 2-year period [8,22]. However, those were qualitative studies using conventional PCR. We showed that the number of JP2 cells was associated with the severity of aggressive periodontitis, making our study the first to reveal a quantitative relationship between the JP2 strain and CAL in aggressive periodontitis. This strongly supports the findings that the *A*. *actinomycetemcomitans* JP2 clone is an agent in the etiology of aggressive periodontitis. However, the quantitative analysis of aggressive periodontitis has just started, and further molecular-based epidemiological studies are required.

Finally, combined with population-based studies, our investigation will contribute to elucidating the quantitative relationship between the number of *A*. *actinomycetemcomitans* JP2 clones and the severity of aggressive periodontitis. These fundamental data will allow risk assessment to determine prevention and treatment strategies for this disease.

## Conclusions

We present the first method for the quantitative detection of *A*. *actinomycetemcomitans* JP2 and non-JP2 clones. The technique is based on real-time PCR methodology with TaqMan probe and primers specifically designed to discriminate these clones. This method was used to quantify *A*. *actinomycetemcomitans* JP2 in the periodontal pockets of patients with aggressive periodontitis, and revealed a significant difference in the number of *A*. *actinomycetemcomitans* JP2 cells between subjects with CALs ≤6 mm and those with CALs ≥7 mm (*p* < 0.01). This technique will contribute to future investigations of the quantitative relationship between *A*. *actinomycetemcomitans* and aggressive periodontitis.

## Methods

### Bacterial strains and culture conditions

The *A*. *actinomycetemcomitans* strains listed in Table 2 were grown in Trypticase soy broth (Becton Dickinson, Sparks, MD) supplemented with 0.6% yeast extract (Becton Dickinson) and 0.04% sodium bicarbonate at 37°C in a 5% CO_2_ atmosphere [34]. Other oral bacteria used in this study are listed as supplemental data (Additional file 2: Table S1).

### DNA techniques

Routine molecular biology techniques were performed as described by Sambrook *et al.*[35]. Chromosomal DNA was isolated from the bacteria listed in Tables 2 and Additional file 2: Table S1 using a Puregene DNA isolation kit (Gentra Systems, Minneapolis, MN). Nucleotide sequence information for *A*. *actinomycetemcomitans* HK1651 was obtained from the Oral Pathogen Sequence Database (Los Alamos National Laboratory, http://www.oralgen.lanl.gov/).

### DNA sequencing of the ∆530 region of *orfX*

The genetic structures of the JP2 clone and non-JP2 strain are shown in Figure 1A. To design highly specific oligonucleotide primers for real-time PCR-based detection of *A*. *actinomycetemcomitans* non-JP2 strains, the ∆530 region of *orfX* was sequenced using two oligonucleotide primer pairs (Table 1). Initially, Aa orfX3'F1 (137–154 bp from the 5’ end of *orfX*) and Aa lktC5'R1 (49–66 bp from the 5’ end of *lktC*) were used for all strains, and Aa orfX3'F2 (33–50 bp from the 5’ end of *orfX*) and Aa lktC5'R2 (37–54 bp from the 5’ end of *lktC*) were used for Aa orfX3'F1- and Aa lktC5'R1-negative strains (Table 2). The PCR conditions consisted of 95°C for 5 min, followed by 25 cycles of 95°C for 30 s, 47°C for 30 s, and 72°C for 1 min for primers Aa orfX3'F1 and Aa lktC5'R1 and 95°C for 5 min, followed by 25 cycles of 95°C for 30 s, 49°C for 30 s, and 72°C for 1 min for primers Aa orfX3'F2 and Aa lktC5'R2. DNA sequencing of these PCR amplicons, performed using an Applied Biosystems 3730 DNA Analyzer (Applied Biosystems, Foster City, CA) and BigDye Terminator v3.1 Cycle Sequence Kit (Applied Biosystems) at Hokkaido System Science (Hokkaido, Japan), yielded a consensus sequence and allowed us to design non-JP2 strain-specific primers (Table 1).

### Primers and probe design

A hydrolysis probe-based qPCR assay (TaqMan assay) was used for the specific detection of *A*. *actinomycetemcomitans* JP2 and non-JP2 strains. The oligonucleotide primers and hydrolysis probe were designed from the *orfX'* region and its 3' border sequences (for JP2 clones; Oral Pathogen Sequence Database number: AA02808) and ∆530 region of *orfX* (for non-JP2 strains) using Primer Express 3.0 (Applied Biosystems). As shown in Table 1, the hydrolysis probe specific for the JP2 clone was labeled with 6-carboxyfluorescein (FAM) and 6-carboxytetramethylrhodamine (TAMRA) and for the non-JP2 clone the hydrolysis probe was labeled with FAM and minor groove binder (MGB). The primer specificity was initially confirmed by gapped blastn 2.0.5 (National Center for Biotechnology Information, http://www.ncbi.nlm.nih.gov/) analysis and then by conventional PCR performed using chromosomal DNA from various oral bacteria (Table 2 and Additional file 2: Table S1). These PCR assays were performed using thermocycler conditions of 94°C for 5 min, followed by 25 cycles of 94°C for 30 s, 60°C for 30 s, and 72°C for 1 min for the JP2 clone and 94°C for 5 min, followed by 25 cycles of 94°C for 30 s, 49°C for 30 s, and 72°C for 1 min for non-JP2 clones.

### qPCR

Chromosomal DNA from bacterial cultures and clinical specimens and a negative control sample were amplified using LightCycler TaqMan Master (Roche Diagnostics GmbH, Mannheim, Germany). The amplification reaction mixture consisted of DNA template, 5× Master Mix, forward and reverse primers (0.5 μM each), 0.2 μM hydrolysis probe, and PCR-grade water in a LightCycler Capillary (Roche Diagnostics). Specific products were amplified and detected using the LightCycler Carousel-based System (Roche Diagnostics), with the following thermal conditions: 95°C for 10 min, followed by 45 cycles of 95°C for 10 s, 58°C for 40 s, and 72°C for 1 s for both JP2 and non-JP2 strains. The endpoint amplification products were further separated on 2% agarose gels to confirm that they were of the predicted sizes.

### Study subjects

Eleven adolescents and young adults 16–31 years old (three males, eight females; mean ± SD age 26.6 ± 4.1 years), diagnosed with aggressive periodontitis at the Dental Hospital, Université Mohammed V Souissi in Rabat, Morocco, participated in this study. Subjects were excluded if they used antibiotics within the preceding year, were undergoing ongoing periodontal treatment (scaling, root planing, and professional tooth cleaning), had orthodontic or prosthetic devices, had used mouth rinse routinely in the previous 6 months, or were smokers.

### Ethics statement

All patients were treated in accordance with the Declaration of Helsinki on the participation of human subjects in medical research. Ethics clearance for the study was obtained from Comité d’Ethique pour la Recherché Biomédicale (CERB), Université Mohammed V Souissi (reference number 400). Patients were fully informed and signed informed consent forms.

### Clinical examination

The clinical attachment level (CAL) in the periodontium was measured as the distance in millimeters from the cement-enamel junction to the bottom of the periodontal pocket using a periodontal probe at eight sites (*i.e.*, the buccal aspects of the mesial surfaces of the upper and lower first incisors and the first molars; if a tooth was missing, the premolar on the same side was used), for a total of 88 sites. To determine the reproducibility of the CAL recordings, all subjects were examined twice at a 2-week interval using identical procedures. The same examiner (O.-K.E.) performed both examinations, but was not aware of the initial findings at the time of the second examination. The reproducibility of recording the CAL was evaluated by measuring the kappa value, which was 0.65.

### Microbiological sampling and preparation

Subgingival samples on paper points were obtained from the adolescents, as described previously [8]. Briefly, subgingival plaque samples were obtained by inserting a sterile paper point into a subgingival site for 10 s. The paper point was transferred into 200 μl of phosphate-buffered saline (PBS) and centrifuged at 12,000 × *g* for 5 min. After centrifugation, the bacteria chromosomal DNA was extracted using MORA-extract (Cosmo Bio, Tokyo, Japan), according to the manufacturer’s instructions, and then served as a template. After centrifugation, the supernatant was removed, and 150 μl of lysis buffer were added to the pellet. The lysed bacteria were transferred to a tube with glass beads and heated at 90°C for 10 min. Then, the bacterial mixture was disrupted using a Mini-Bead Beater (Biospec Products, Bartlesville, OK) with 0.1-mm-diameter glass beads at 4,800 rpm for 2 min. After this procedure, 200 μl of SDS solution were added and heated at 90°C for 10 min. Then, 400 μl of phenol solution were added and mixed for 1 min. After centrifugation, the aliquot was subjected to ethanol precipitation and dissolved in 20 μl of TE buffer.

### Statistical analysis

Intergroup bacterial cell numbers were analyzed using the Mann–Whitney test. The kappa value was measured to determine the reproducibility of recording the CAL. Statistical analysis was performed with the program SPSS 11.0 (SPSS, Chicago, IL), and the differences were considered significant when the *p*-value was less than 0.05.

## Abbreviations

qPCR: Quantitative polymerase chain reaction; CAL: Clinical attachment level; CFU: Colony forming unit; Ct: Threshold cycle; FAM: 6-carboxy-fluoresein; TAMRA: 6-carboxy-tetramethylrhodamine; MGB: Minor groove binder.

## Competing interests

The authors declare that they have no competing interests.

## Authors’ contributions

AY participated in the design of the study, perfected the DNA manipulations, performed qPCR analyses, wrote the first draft of the manuscript, and was responsible for the overall coordination of the study. O-KE performed the clinical examinations and samplings of subgingival plaque at a dental hospital in Rabat, Morocco. HM provided the contacts with the staff of the Faculté de Médecine Dentaire, Université Mohammed V Souissi, Rabat, Morocco. TH and HH organized and performed the microbiological studies. TN organized and supervised the microbiological studies. TA and SA conducted the statistical analyses. All authors collaboratively interpreted the results, were involved in writing the manuscript, and approved the final version.

## Pre-publication history

The pre-publication history for this paper can be accessed here:

http://www.biomedcentral.com/1471-2334/12/253/prepub

## Supplementary Material

Additional file 1 **Table S2.** Homology analysis of the Δ530 region of *A*. *actinomycetemcomitans* non-JP2 strains. (PDF 57 kb) Click here for file

Additional file 2 **Table S1.** Bacterial strains other than *Aggregatibacter actinomycetemcomitans* used for primer-specificity analysis. (PDF 12 kb) Click here for file
