# Supplementary material for: Quantitative discrimination of Aggregatibacter actinomycetemcomitans highly leukotoxic JP2 clone from non-JP2 clones in diagnosis of aggressive periodontitis
Source: BMC Infect Dis. 2012 Oct 11;12:253. doi: 10.1186/1471-2334-12-253 (PMC3523965; doi:10.1186/1471-2334-12-253)
Supplement: Additional file 1 — Table S2. Homology analysis of the Δ530 region of A. actinomycetemcomitans non-JP2 strains. (PDF 57 kb) [file 1471-2334-12-253-S1.pdf]

TABLE S2. Homology analysis of the Δ530 region of *A. actinomycetemcomitans* non-JP2 strains.

Strain (serotype)	ATCC 29523 (a)	SUNYaB 75 (a)	TN-1 (a)	[a]Y4 (b)	HK 1709 (b)	[a]ATCC 29522 (b)	ATCC 29524 (b)	HK 916 (c)	SUNYaB 67 (c)	NCTC 9709 (c)	3381 (d)	IDH 781 (d)	OMZ 534 (e)	OMZ 541 (e)	OMZ 546 (e)	NUM 5505 (f)	NUM 4039 (g)
ATCC 29523 (a)	100	100	100	98.682	100	98.493	99.435	99.812	98.682	100	100	98.483	99.435	99.247	97.363	100	100
SUNYaB 75 (a)	100	100	100	98.682	100	98.493	99.435	99.812	98.682	100	100	98.493	99.435	99.247	97.363	100	100
TN-1 (a)	100	100	100	98.682	100	98.493	99.345	99.812	98.682	100	100	98.493	99.435	99.247	97.363	100	100
[a]Y4 (b)	98.682	98.682	98.682	100	98.682	99.811	98.87	98.493	100	98.682	98.682	97.175	99.247	99.058	97.175	98.682	98.682
HK1709 (b)	100	100	100	98.682	100	98.493	98.435	99.812	98.682	100	100	98.493	99.435	99.247	97.363	100	100
[a]ATCC 29522 (b)	98.493	98.493	98.493	99.811	98.493	100	98.682	98.305	99.811	98.493	98.493	96.987	99.058	98.87	96.987	98.493	98.493
ATCC 29524 (b)	99.435	99.435	99.435	98.87	99.435	98.682	100	99.247	98.87	99.435	99.435	97.928	99.623	99.435	97.552	99.435	99.435
HK 916 (c)	99.812	99.812	99.812	98.493	99.812	98.305	99.247	100	98.493	99.812	99.812	98.305	99.247	99.058	97.175	99.812	99.812
SUNYaB 67 (c)	98.682	98.682	98.682	100	98.682	99.811	98.87	98.493	100	98.682	98.682	97.175	99.247	99.058	97.175	98.682	98.682
NCTC 9709 (c)	100	100	100	98.682	100	98.493	99.435	99.812	98.682	100	100	98.493	99.435	99.247	97.363	100	100
3381 (d)	100	100	100	98.682	100	98.493	99.345	99.812	98.682	100	100	98.493	99.435	99.247	97.363	100	100

IDH 781 (d)	98.493	98.493	98.493	97.175	98.493	96.978	97.928	98.305	97.175	98.493	98.493	100	97.928	97.74	98.493	98.493	98.493
OMZ534 (e)	99.435	99.435	99.435	99.247	99.435	99.058	99.623	99.247	99.247	99.435	99.435	97.928	100	99.821	97.928	99.435	99.435
OMZ541 (e)	99.247	99.247	99.247	99.058	99.247	98.87	99.435	99.058	99.058	99.247	99.247	97.74	99.821	100	97.740	99.247	99.247
OMZ546 (e)	97.363	97.363	97.363	99.175	97.363	96.987	97.552	97.175	97.175	97.363	97.363	98.493	97.928	97.74	100	97.363	97.363
NUM5505 (f)	100	100	100	98.682	100	98.493	99.345	99.812	98.682	100	100	98.493	99.435	99.247	97.363	100	100
NUM4039 (g)	100	100	100	98.682	100	98.493	99.345	99.812	98.682	100	100	98.493	99.435	99.247	97.363	100	100

[a]Δ530 region are consisted of 528 bp and all other strains are consisted of 531 bp.
