# Supplementary material for: Quantitative discrimination of Aggregatibacter actinomycetemcomitans highly leukotoxic JP2 clone from non-JP2 clones in diagnosis of aggressive periodontitis
Source: BMC Infect Dis. 2012 Oct 11;12:253. doi: 10.1186/1471-2334-12-253 (PMC3523965; doi:10.1186/1471-2334-12-253)
Supplement: Additional file 2 — Table S1. Bacterial strains other than Aggregatibacter actinomycetemcomitans used for primer-specificity analysis. (PDF 12 kb) [file 1471-2334-12-253-S2.pdf]

TABLE S1. Bacterial strains other than *Aggregatibacter actinomycetemcomitans* used for primer-specificity analysis.

Porphyromonas gingivalis W83
Porphyromonas gingivalis ATCC 33277
Treponema denticola ATCC 31211
Prevotella intermedia ATCC 25611
Prevotella nigrescens ATCC 25261
Prevotella melaninogenica ATCC 25845
Prevotella loescheii ATCC 15930
Prevotella corporis ATCC 33547
Prevotella oralis ATCC 33322
Prevotella veroralis ATCC 33779
Tannerella forsythia ATCC 43037
Fusobacterium nucleatum ATCC 10953
Haemophilus aphrophilus NCTC 5980
Streptococcus mutans Xc
Streptococcus mutans UA159
Streptococcus sobrinus OMZ176
Streptococcus sanguinis OMZ9
Streptococcus gordonii DL1
Streptococcus oralis ATCC 10557
Lactobacillus
Actinomyces viscosus ATCC 15988
Escherichia coli DH5α
